# Wet-Bulb Temperature as a Superior Predictor of Milk Yield and Reproductive Performance in Holstein Cows in a Continental Climate

**DOI:** 10.3390/vetsci13020149

**Published:** 2026-02-04

**Authors:** Onur Erzurum, Tamer Kayar

**Affiliations:** 1 Department of Veterinary, Karapinar Aydoganlar Vocational School, Selcuk University, Konya 42130, Türkiye; 2 Department of Animal Science, Faculty of Veterinary Medicine, Aksaray University, Aksaray 68100, Türkiye; tamerkayar@aksaray.edu.tr

**Keywords:** Holstein, heat stress, wet-bulb temperature (T_wb_), milk yield, dry heat, THI

## Abstract

This study analyzed how climate variability affects lactation yield and reproduction in Holstein cows in a continental climate. It specifically compared Wet-Bulb Temperature (T_wb_) with the standard Temperature–Humidity Index (THI). We conducted a retrospective study on a dairy farm in Konya, Türkiye, analyzing a total of 144 complete lactation records from a herd of 90 cows calving between 2022 and 2023. To rule out nutritional factors, a consistent TMR diet without pasture access was maintained in both years. Average Daily Milk Yield (ADMY) was calculated to adjust for lactation length. Climatic data showed a distinct contrast. Ambient temperatures and THI were similar between years (*p* > 0.05). However, 2022 was characterized by “humid heat” (high T_wb_), while 2023 presented a “dry heat” profile with significantly lower T_wb_ (*p* < 0.001). This difference significantly impacted performance. Cows in the 2023 group produced much higher milk yields (50.55 ± 3.01 kg) than the 2022 group (30.74 ± 0.81 kg) (*p* < 0.001). Unexpectedly, milk yield peaked during the Autumn and Summer seasons of the low-humidity year. In contrast, fertility declined with thermal load. Poor winter fertility suggested a “carry-over” effect of previous heat stress. These findings show that T_wb_ is a better indicator of thermal comfort than THI in continental climates. Furthermore, low humidity can significantly reduce the negative impact of heat on milk production.

## Simple Summary

This study compared heat stress indicators for Holstein cows in a continental climate. We analyzed milk production over two years. One year was “humid” and the other was “dry”. Although air temperatures were similar, cows produced significantly more milk during the dry year. This is because dry air allows cows to cool down more effectively. The standard Temperature–Humidity Index (THI) failed to explain this difference. However, the Wet-Bulb Temperature (T_wb_) accurately predicted the higher milk yield. We also found that summer heat stress caused delayed fertility problems in the winter. Therefore, we recommend using T_wb_ instead of THI to manage heat stress in dry regions.

## 1. Introduction

Global climate change has intensified thermal challenges in livestock production systems, particularly affecting dairy cattle performance in many regions. Fluctuations in ambient temperature have become a significant concern affecting livestock productivity, particularly in dairy production systems [1]. Increased environmental temperatures can cause heat stress in dairy cows, adversely affecting both milk production and reproductive performance [2,3,4,5]. The early stage of lactation, particularly the first 60 days, is a critical window where cows are especially sensitive to thermal load [6,7]. Throughout the entire lactation period, heat stress may lead to a 10–25% reduction in milk yield and a weakened immune system [5].

Understanding the environmental drivers of heat stress is therefore essential to mitigate its impact on dairy productivity. In dairy cattle, heat stress results from the combined effects of various environmental factors, such as temperature, relative humidity, and wind speed. Relative humidity (RH) denotes the proportion of moisture in the air [8]. The temperature–humidity index (THI) is commonly used to quantify the level of heat stress. The thermal tolerance of animals varies by species [9].

A critical limitation of standard THI formulations is their heavy reliance on dry-bulb temperature (T_db_), often underestimating the impact of atmospheric moisture in certain climatic conditions. While THI is widely used, it may not fully capture the evaporative cooling potential of the environment, a crucial mechanism for high yielding cows to dissipate metabolic heat [10]. This is where alternative indices, such as Wet-Bulb Temperature (T_wb_), become relevant. T_wb_, measured using a thermometer wrapped in a water-soaked cloth, directly reflects the cooling efficiency achievable through evaporation. Since thermal sensitivity involves a complex interplay of temperature and humidity, relying solely on THI may mask the true thermal load, especially in regions with significant inter-annual humidity variations [8].

However, there is limited research integrating seasonal thermal indices—such as T_wb_ and dew point—with calving time and subsequent milk yield. Several studies have investigated the effects of temperature during the calving period on subsequent lactation performance [11,12,13,14,15]. Among the physiological stages of dairy cows, the calving period represents a critical window during which thermal stress may have long-term impacts on productivity. Therefore, the objective of this study was to evaluate the influence of climatic variability at calving on the lactation performance of Holstein cows and to determine whether dynamic thermal indices, particularly T_wb_, offer a more accurate prediction of milk yield and reproductive performance than standard metrics under varying humidity conditions.

## 2. Material and Methods

### 2.1. Study Area

The study was conducted using retrospective herd data from a commercial dairy farm located in Konya Province, Türkiye, characterized by a continental climate. Ethical approval for the study was obtained from the Ethics Committee of Selcuk University, Faculty of Veterinary Medicine, Experimental Animal Production and Research Center (SUVDAMEK) with decision number 2025/51.

### 2.2. Animals

The study population comprised 90 Holstein cows maintained throughout the two-year study period (2022–2023). Due to the multi-year duration of the study, a total of 144 complete lactation records were available for analysis. The selection criterion focused on continuous healthy status to minimize the confounding effects of genetic turnover. The lactation numbers (parity) of the analyzed records ranged from 2 to 7. The herd composition was predominantly centered on early-to-mid parities, with only two cows recorded at the 7th parity.

### 2.3. Herd Management and Feeding Strategies

To ensure that the observed variations in milk yield were primarily driven by climatic factors rather than nutritional or management changes, a consistent intensive management system was maintained throughout the study period (2022–2023).

The animals were housed in a free-stall barn system equipped with rubber mats and automatic waterers. The barn relied on natural ventilation; no active cooling strategies (fans/sprinklers) were employed. The cows did not have access to pasture grazing at any time. However, to support animal welfare, the cows were provided access to an adjacent open-air exercise area (paddock) with compost bedding during the warmer months (from late Spring to early Winter). This area was strictly bounded and did not offer any grazing material, ensuring that nutrient intake remained controlled.

A Total Mixed Ration (TMR)-based feeding program was implemented year-round. The ration was formulated to meet the nutritional requirements of high-producing Holstein cows according to NRC (National Research Council) standards. Crucially, the feed composition and nutritional density (energy and protein levels) remained standardized across both 2022 and 2023. The TMR was delivered twice daily, and cows had ad libitum access to feed and fresh water. No significant changes in the ration formulation, feed additives, or feeding frequency occurred between the two study years, thereby minimizing the confounding effect of nutrition on the inter-annual yield comparison.

### 2.4. Variable Calculation and Climate Data

Data were extracted from the farm’s computerized herd management system. Primary variables included: Calving Date, Total Lactation Yield (kg), Days in Milk (*DIM*), Lactation Number, and Number of Inseminations per Conception.

To eliminate the confounding effect of lactation length variations on production analysis, Average Daily Milk Yield (ADMY) was calculated for each cow using the following formula:

$$ADMY(\frac{kg}{day})=\frac{\left(\mathrm{Total}\;\mathrm{Lactation}\;\mathrm{Yield}\right)}{DIM}$$

where ADMY is average daily milk yield (kg), Total Lactation Yield is the total milk produced in the lactation (kg), and *DIM* is days in milk.

ADMY was selected to reflect the actual daily physiological response to the thermal environment, avoiding the projection bias of standardized formulas. Instances of shorter lactations were observed to be randomly distributed between the years, ensuring no systematic bias in the inter-annual comparison.

Raw meteorological data (ambient temperature and relative humidity) were obtained on a monthly basis from the Turkish State Meteorological Service (MGM) for the years 2022 and 2023. These values were used to calculate the specific thermal indices using the equations detailed in the following section. Calving months were grouped into four seasons based on the calendar year: Winter (January, February, and December of the respective year), Spring (March–May), Summer (June–August), and Autumn (September–November). Crucially, to capture inter-annual climate variability, each cow was assigned the specific thermal index values corresponding to her specific month and year of calving.

### 2.5. Calculation of Thermal Indices

Three thermal indices were calculated to evaluate environmental stress loads:

Temperature–Humidity Index (THI) calculated using the equation by Yousef [9]:

$$THI=T_{db}+\left(0.36\times T_{dp}\right)+41.2$$

where T_db_ is dry-bulb temperature (°C) and T_dp_ is dew point temperature (°C).

Dew Point Temperature (T_dp_) calculated according to Lawrence [16]:

T_dp_ = T − (100 − RH)/5

where T is ambient temperature (°C) and RH is relative humidity (%).

Wet-Bulb Temperature (T_wb_) calculated using the Stull [17] formula, which incorporates arctangent functions of temperature and humidity to estimate evaporative cooling potential.

T_wb_ = T × arctan (0.151977 × (RH + 8.313659)0.5) + arctan (T + RH) − arctan (RH − 1.676331) + 0.00391838 × RH1.5 × arctan (0.023101 × RH) − 4.686035

where T is dry-bulb temperature (°C) and RH is relative humidity (%).

Heat stress levels were categorized based on THI values according to the classification system proposed by Moretti et al. [18], as presented in Table 1. This classification was selected as it offers higher sensitivity for detecting heat stress in modern high-yielding dairy cows compared to older traditional standards.

### 2.6. Statistical Analysis

Statistical analyses were performed using SPSS software (version 29). Prior to analysis, the normality of data distribution was assessed using the Kolmogorov–Smirnov test. Due to significant climatic differences observed between the two years, data from 2022 and 2023 were analyzed separately to elucidate the differential effects of dry and humid heat.

Comparison of Years: Independent samples *t*-test was used to compare climatic parameters (THI, T_wb_) and production traits between 2022 and 2023 (Table 2).

Seasonal Analysis: One-way Analysis of Variance (ANOVA) was employed to evaluate the effect of calving season on ADMY and reproductive performance. Post hoc comparisons were performed using Tukey’s test.

Correlation Analysis: Pearson correlation coefficients were calculated using the total dataset of individual lactation records (*n* = 144) to assess the relationships between thermal indices (THI, T_wb_) and production traits. Descriptive statistics are presented as Mean and pooled Standard Error. The significance level was set at *p* < 0.05.

## 3. Results

### 3.1. Inter-Annual Climate Variability

A distinct climatic contrast was observed between the two study years. Although the mean Ambient Temperature (T) and THI values were comparable between 2022 and 2023 (*p* > 0.05), a highly significant difference was recorded in T_wb_ (Table 2). The year 2022 was characterized by significantly higher humidity and T_wb_ values (10.51 ± 0.95 °C), indicating a “humid heat” profile. In contrast, 2023 presented a “dry heat” profile with significantly lower T_wb_ values (5.45 ± 0.65 °C) (*p* < 0.001) (Table 2).

**Figure 1:**
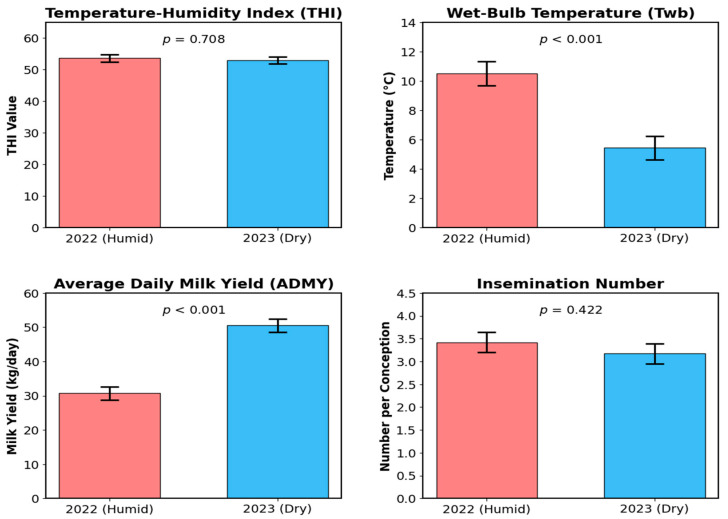
Comparison of climatic parameters (THI, T_wb_) and production traits (ADMY, Insemination Number) between the humid (2022) and dry (2023) years. Bars represent Mean ± Pooled SE. *p*-values indicate statistical significance between years.

Cows calving in 2023, benefitting from lower T_wb_ and better evaporative cooling potential, achieved significantly higher Average Daily Milk Yield (ADMY) compared to the year 2022 (*p* < 0.001) (Table 2, Figure 1).

### 3.2. Effect of Calving Season on Performance

**Figure 2:**
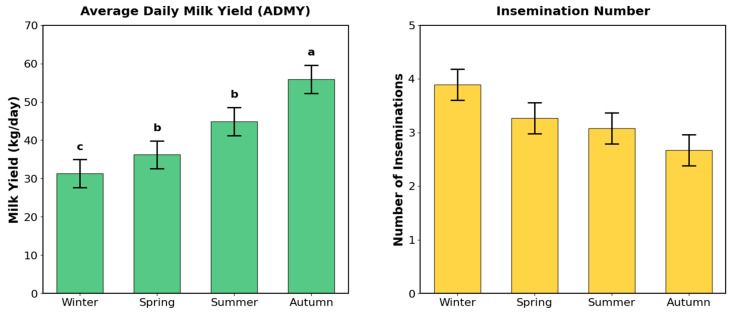
Seasonal dynamics of Average Daily Milk Yield (ADMY) and Insemination Number. Bars represent Mean ± Pooled SE. Different letters (a, b, c) indicate significant differences for ADMY (*p* < 0.001); no significant differences were found for Insemination Number (*p* = 0.113) (Table 3).

As shown in Table 3, there was a statistically significant difference in ADMY among seasons (F_(3-140)_ = 17.23, *p* < 0.001). Scheffe post hoc test results indicated that the highest milk yield was observed in the autumn season, which differed significantly from the other seasons (Figure 2).

Conversely, no statistically significant difference was found among seasons regarding the number of inseminations (F_(3-140)_ = 2.03, *p* = 0.113) (Table 3).

### 3.3. Relationship Between Thermal Indices and Production

Pearson correlation analysis (Table 4) highlighted the complex relationship between thermal load and milk yield. When data from both years were combined, a positive correlation was observed between thermal indices (THI, T_wb_) and ADMY.

Conversely, a negative correlation trend was observed between thermal indices and reproductive efficiency (Insemination Number).

## 4. Discussion

### 4.1. The Critical Role of Wet-Bulb Temperature in Yield Variability

This study focused on the comparative accuracy of thermal indices. Addressing this objective, our most striking finding is the decoupling of milk yield from standard THI values and its strong dependence on T_wb_. Crucially, feeding and management practices remained constant throughout the two-year period. Consequently, the observed fluctuations in milk yield are largely attributable to environmental variances. Furthermore, the herd remained genetically stable with no recorded disease outbreaks between the years. This minimizes the impact of non-climatic confounding factors.

While ambient temperatures and THI were statistically similar between 2022 and 2023 (*p* > 0.05), the milk yield in 2023 was significantly higher. This contradicts earlier generalizations that ambient temperature is the primary driver of production losses [3]. Instead, our results align with the specific climatic nuances described by Bohmanova et al. [19]. They demonstrated that in dry climates, indices heavily weighted on humidity (like standard THI) might misrepresent the actual thermal load.

The year 2023 was characterized by a “dry heat” profile with significantly lower T_wb_ (5.45 °C) compared to the “humid heat” of 2022 (10.51 °C). T_wb_ represents the thermodynamic limit of evaporative cooling [17]. Physiologically, dairy cattle rely heavily on cutaneous evaporation (sweating) and respiratory heat loss (panting) to maintain homeostasis [3,20]. These cooling mechanisms depend entirely on the vapor pressure gradient between the animal and the air. Standard THI often overlooks this gradient. However, T_wb_ directly reflects the potential for moisture evaporation. Consequently, the lower T_wb_ in 2023 maximized this gradient. This allowed high-yielding cows to dissipate metabolic heat more efficiently. This confirms that evaporative potential is a more critical determinant of performance than dry-bulb temperature alone in continental climates [3,19]. This finding aligns with recent research by Jo and Lee [21]. They demonstrated that high relative humidity significantly worsens heat stress in early-lactation Holsteins, leading to reduced milk yield and increased cortisol levels. Consequently, the lower humidity in 2023 minimized this physiological burden, providing a clear productive advantage.

### 4.2. Seasonal Dynamics and the “Dry Heat” Advantage

Contrary to the widespread consensus that milk yield is lowest during summer due to heat stress [2,12], our study observed the highest ADMY in cows calving during the Autumn season, followed by the Summer season. This unexpected finding can be explained by the specific interaction between the herd’s lactation curve and the “dry heat” phenomenon of 2023 [19].

The literature indicates that high relative humidity exacerbates the effects of heat stress by limiting heat dissipation [1]. However, the 2023 cohort, which included high-genetic-merit cows, peaked during a period of low T_wb_. This suggests that Holsteins can sustain high production levels even at elevated temperatures, provided the air is dry enough to facilitate efficient evaporation. This observation parallels findings by Correa-Calderon et al. [22], who noted that cooling management effectiveness is maximized under dry conditions, thereby mitigating the expected summer production slump.

### 4.3. Reproductive Performance and the “Carry-Over” Effect

A notable finding of this study was the pattern of reproductive performance. No statistically significant difference was observed in the number of inseminations per conception among seasons (*p* > 0.05). Contrary to the assumption that winter always offers optimal reproductive conditions, fertility during the winter season did not differ significantly from the summer season.

This phenomenon is best explained by the “carry-over effect” of heat stress described by Roth et al. [23] and Hansen [4]. The deleterious effects of hyperthermia are not limited to the acute phase of stress. Heat stress impairs the developmental competence of oocytes residing in pre-antral follicles [23,24]. The developmental trajectory from the pre-antral to the pre-ovulatory stage spans approximately 60 to 80 days [25]. Consequently, follicles exposed to thermal stress during the preceding late summer and autumn months complete their maturation and are ovulated in winter. These oocytes often exhibit reduced fertilization capacity or lead to early embryonic death. Thus, the poor winter performance observed in our study reflects a delayed manifestation of thermal damage accumulated during the previous season.

### 4.4. Methodological Implications: Superiority of T_wb_

Standard THI formulas were originally developed for humans [26] or cattle in specific climates [27] and often prioritize dry-bulb temperature. While recent studies continue to demonstrate the utility of THI in tropical regions [28], our 2022–2023 comparison clearly demonstrates the limitations of THI in distinguishing between “humid heat” and “dry heat” years. While THI failed to predict the yield difference, T_wb_ successfully captured the environmental advantage of 2023.

Therefore, for dairy operations in regions with significant humidity fluctuations (like Central Anatolia), we propose that T_wb_ should be integrated into heat stress monitoring protocols. As suggested by Gaughan et al. [29], using multidimensional indices that account for evaporative potential provides a more accurate basis for activating cooling systems (e.g., fans and sprinklers) than temperature-based thresholds alone.

### 4.5. Limitations and Future Perspectives

The single-farm design allowed for strict control over nutritional and management variables. However, this approach limits the generalization of findings to other regions or systems. We acknowledge that the specific microclimate of the selected farm likely influenced the observed effects. While this study utilized monthly data to reveal broad seasonal trends, future research incorporating daily or hourly climatic variations would provide higher resolution to capture short-term heat stress events. Therefore, future research should validate the superiority of T_wb_ using larger, multi-farm datasets. Additionally, prospective studies should include physiological markers, such as respiration rate, to further explain the biological mechanisms behind the “dry heat” advantage. Finally, due to the retrospective nature of the study, data regarding milk composition and detailed reproductive intervals were unavailable for analysis. However, recent studies have highlighted that heat stress significantly alters milk fatty acid profiles and quality parameters [30], suggesting this as a critical area for future investigation in continental climates.

## 5. Conclusions

The primary objective of this study was to identify accurate predictors of performance under varying humidity. Fulfilling this aim, our results provide compelling evidence that T_wb_ is a superior indicator of thermal comfort compared to the standard THI for dairy cattle in continental climates. Our inter-annual analysis revealed that while ambient temperatures were similar across years, the specific humidity profile played a decisive role in production outcomes. Crucially, the standardization of nutritional and management strategies throughout the study period allowed for the isolation of these environmental effects, eliminating potential confounding factors. The “dry heat” conditions of 2023, characterized by significantly lower T_wb_ values, allowed high-yielding Holstein cows to maintain superior milk production levels compared to the “humid heat” conditions of 2022, likely due to enhanced evaporative cooling efficiency.

Contrary to traditional expectations, milk yield was maximized during the Autumn seasons in the low-humidity cohort, suggesting that dairy breeds can sustain high performance in warmer months if the moisture load is managed effectively.

These findings suggest that dairy management strategies in Central Anatolia and similar regions should transition from relying solely on dry-bulb temperature or standard THI to incorporating T_wb_ into heat stress monitoring protocols. Implementing cooling interventions based on evaporative potential rather than just temperature thresholds may offer a more precise approach to optimizing both lactation and fertility under changing climate conditions.

## Figures and Tables

**Table 1:** Threshold classification of temperature–humidity index (THI) for heat stress evaluation.

Class	THI Range	Condition
1	<68	Safe
2	68 ≤ THI ≤ 72	Mild discomfort
3	72 ≤ THI ≤ 75	Discomfort
4	75 ≤ THI ≤ 79	Alert
5	79 ≤ THI ≤ 84	Danger
6	THI ≥ 84	Emergency

**Table 2:** Comparison of climatic parameters and production traits between 2022 and 2023.

Parameter	2022 (Humid Year)	2023 (Dry Year)	SEM	*p*
THI	53.64	53.03	1.15	0.708
T_wb_ (°C)	10.51	5.45	0.81	<0.001
ADMY (kg/day)	30.74	50.55	1.95	<0.001
Insemination Number	3.42	3.17	0.22	0.424

ADMY: Average Daily Milk Yield; THI: Temperature–Humidity Index; T_wb_: Wet-Bulb Temperature; SEM: Pooled Standard Error of the Mean.

**Table 3:** Effect of calving season on average daily milk yield (ADMY) and insemination number.

Season	N	ADMY (kg/Day)	Insemination Number
Autumn	33	55.91 ^a^	2.67
Summer	35	44.91 ^b^	3.08
Spring	48	36.21 ^b^	3.27
Winter	28	31.33 ^c^	3.89
Statistics			
SEM		3.65	0.29
F-value		17.23	2.03
*p*-value		<0.001	0.113

SEM: Pooled Standard Error of the mean. ^a, b, c^: Means within the same column with different superscripts differ significantly (*p* < 0.05). ADMY: R = 0.519, R^2^ = 0.270; Insemination number: R = 0.204, R^2^ = 0.042.

**Table 4:** Pearson correlation coefficients between thermal indices and production traits.

Variable	ADMY (kg)	Insemination Number
THI	r = 0.27 (*p* < 0.01)	r = −0.17 (*p* < 0.05)
T_wb_	r = 0.28 (*p* < 0.01)	r = −0.16 (*p* < 0.05)

r = Pearson correlation coefficient. ADMY = Average Daily Milk Yield; T_wb_ = Wet-Bulb Temperature.

## Data Availability

The original contributions presented in this study are included in the article. Further inquiries can be directed to the corresponding author.
